# Molecular Testing for Fragile X: Analysis of 5062 Tests from 1105 Fragile X Families—Performed in 12 Clinical Laboratories in Spain

**DOI:** 10.1155/2014/195793

**Published:** 2014-05-28

**Authors:** María-Isabel Tejada, Guillermo Glover, Francisco Martínez, Miriam Guitart, Yolanda de Diego-Otero, Isabel Fernández-Carvajal, Feliciano J. Ramos, Concepción Hernández-Chico, Elizabet Pintado, Jordi Rosell, María-Teresa Calvo, Carmen Ayuso, María-Antonia Ramos-Arroyo, Hiart Maortua, Montserrat Milà

**Affiliations:** ^1^Laboratorio de Genética Molecular, Servicio de Genética, Hospital Universitario Cruces, BioCruces Health Research Institute, GCV-CIBER de Enfermedades Raras (CIBERER-ISCIII), Barakaldo, 48903 Bizkaia, Spain; ^2^Unidad de Genética Molecular, Centro de Bioquímica y Genética Clínica, Hospital Clínico Universitario Virgen de la Arrixaca, El Palmar, 30120 Murcia, Spain; ^3^Unidad de Genética, Hospital Universitario La Fe, 46009 Valencia, Spain; ^4^Laboratorio de Genética, UDIAT-Centre Diagnòstic, Corporació Sanitària Parc Taulí, Institut Universitari UAB, Sabadell, 08208 Barcelona, Spain; ^5^Unidad de Gestión Clínica de Salud Mental, Hospital Regional Universitario de Málaga, Instituto de Investigación Biomédica de Málaga (IBIMA), 29010 Málaga, Spain; ^6^Instituto de Biología y Genética Molecular (IBGM), Universidad de Valladolid, CSIC, 47003 Valladolid, Spain; ^7^Consulta de Genética Clínica, Servicio de Pediatría, Hospital Clínico Universitario Lozano Blesa, Facultad de Medicina, Universidad de Zaragoza, GCV-CIBER de Enfermedades Raras (CIBERER-ISCIII), 50009 Zaragoza, Spain; ^8^Servicio de Genética, Hospital Ramón y Cajal, 28034 Madrid, Spain; ^9^Servicio de Biología Molecular, Hospital Virgen Macarena y Universidad de Sevilla, 41009 Sevilla, Spain; ^10^Servicio de Genética, Hospital Universitari Son Espases, GCV-CIBER de Enfermedades Raras (CIBERER-ISCIII), Palma de Mallorca, 07010 Illes Balears, Spain; ^11^Unidad de Genética Médica, Hospital Universitario Miguel Servet, 50009 Zaragoza, Spain; ^12^Servicio de Genetica, IIS-Hospital Universitario Fundación Jiménez Díaz (IIS-FJD, UAM), CIBER de Enfermedades Raras (CIBERER-ISCIII), 28040 Madrid, Spain; ^13^Servicio de Genética, Complejo Hospitalario de Navarra, 31008 Pamplona, Spain; ^14^Servicio de Bioquímica y Genética Molecular, Hospital Clinic, IDIBAPS, CIBER de Enfermedades Raras (CIBERER-ISCIII), 08036 Barcelona, Spain

## Abstract

Fragile X syndrome is the most common inherited form of intellectual disability. Here we report on a study based on a collaborative registry, involving 12 Spanish centres, of molecular diagnostic tests in 1105 fragile X families comprising 5062 individuals, of whom, 1655 carried a full mutation or were mosaic, three cases had deletions, 1840 had a premutation, and 102 had intermediate alleles. Two patients with the full mutation also had Klinefelter syndrome. We have used this registry to assess the risk of expansion from parents to children. From mothers with premutation, the overall rate of allele expansion to full mutation is 52.5%, and we found that this rate is higher for male than female offspring (63.6% versus 45.6%; *P* < 0.001). Furthermore, in mothers with intermediate alleles (45–54 repeats), there were 10 cases of expansion to a premutation allele, and for the smallest premutation alleles (55–59 repeats), there was a 6.4% risk of expansion to a full mutation, with 56 repeats being the smallest allele that expanded to a full mutation allele in a single meiosis. Hence, in our series the risk for alleles of <59 repeats is somewhat higher than in other published series. These findings are important for genetic counselling.

## 1. Introduction

Fragile X syndrome (FXS) is the most common cause of inherited intellectual disability (ID) [1]. It is an X-linked dominant disease with incomplete penetrance, affecting approximately 1/3717 to 1/8918 Caucasian males [2]. Although rare, it is one of the most prevalent genetic disorders; this is the reason for its medical and social importance.

In affected males, the syndrome is characterised by moderate-to-severe mental retardation with behavioural disturbances such as hyperactivity and stereotypic hand flapping in addition to variable dysmorphic features such as large everted ears, elongated face, and postpubertal macroorchidism [1]. Affected females tend to have milder symptoms of FXS than males and they rarely show physical features.

The* FMR1* gene was identified in 1991, and FXS is now known to be caused by the anomalous expansion of a trinucleotide CGG repeat located in the 5′ end of this gene at the FRAXA locus in Xq27.3 [3]. The number of CGG repeats in the population has been classified into four groups depending on repeat size: normal (N), with 6 to 44 repeats; intermediate (IA)—also called “grey alleles”—with between 45 and 54 CGG repeats; premutation (PM) with between 55 and 200 repeats; and the so-called full mutation (FM) with over 200 repeats [4]. In the last group, a second mechanism is triggered, the hypermethylation of the adjacent CpG island, resulting in a shutdown of transcription and therefore lack of production of the FMRP protein, which is the underlying cause of the syndrome [5].

The term “premutation” was coined to reflect the fact that PM carriers do not generally have ID but that their alleles are usually unstable, resulting in an expansion of the CGG repeats when transmitted by a female and, hence, offsprings of female PM carriers are at risk of having FXS [3]. The* FMR1* PM affects both males and females and it seems that as many as 1/130–260 females and 1/250–810 males are carriers of a PM [6]. In recent years, it has been seen that IAs may or may not be unstable [7]. It has also been demonstrated that the risk of expansion is related to the number of CGG repeats, with smaller alleles being less likely to expand to a full mutation than larger ones [5, 8]. The smallest premutation that has been reported to expand to a full mutation allele in one generation had 59 CGG repeats [7].

It was initially thought that females with a PM were completely asymptomatic, but it was soon realized that this is not the case: in 1996, a family was described in which the women with a PM presented with clinical symptoms seemingly unrelated to ID: a premature ovarian failure leading to premature menopause [9]. Over the years these findings have been confirmed in numerous studies, all pointing to fragile X-associated primary ovarian insufficiency (FXPOI), as a phenotypic characteristic of PM carriers although only about 13–26% of them present with this trait [10]. Interestingly, full mutation carriers do not seem to present FXPOI. Furthermore, PM alleles are associated with a significant elevation of* FMR1* mRNA levels [11, 12] and it has been shown that carriers of the* FMR1* premutation are at risk of developing fragile X-associated tremor/ataxia syndrome (FXTAS), a late-onset neurodegenerative disorder usually affecting males over 50 years of age [13]. In short, fragile X (FX) is now understood to be a family of disorders including FXS, FXPOI, and FXTAS [1].

In Spain, the frequency of the FM alleles has been estimated to be approximately 1 in 2633 [14] and 1 in 2466 [15] in two different studies in male newborns, and the disease prevalence has been estimated to be 1 : 5000–1 : 6800 in males [16]. However, the real number of individuals with FM or PM remains unknown, not only in Spain, but also in other countries. Furthermore, clinical features are neither specific nor constant in carriers of the FM or the PM and, hence, the exact frequencies of all of these types of clinical involvement remain unknown in most of the populations studied.

With the overall aim of adding our knowledge to what is already known about this syndrome, we created a Fragile X Registry so that we could use it to improve diagnosis, prevention, and genetic counselling in these families. Thanks to the collaboration of 12 clinical laboratories with members of GIRMOGEN (a Spanish Network for the study of intellectual disabilities of genetic origin) that have received samples from patients all over Spain, we have collected clinical and molecular information from 19 years of diagnostic work on a large number of members of FX families. In this work, we present the first part of the statistical analysis of this data to provide new information to guide clinical practice, specifically concerning the clinical indications, molecular results, and transmission of the expansions for the purposes of genetic counselling. Despite the large series already published [7, 8, 14, 15, 17–19], this work is one of the few including individuals within FX families; this is the reason for the importance of this report.

## 2. Materials and Methods

### 2.1. Patients

Data from FX patients and their direct relatives were retrospectively collected from clinical laboratories at 12 diagnostic reference centres of several Spanish regions.  Table 1 lists these centres and numbers of cases and families recruited. The total number of individuals studied includes all index cases (ICs) plus all their relatives, including prenatal cases. We considered an IC as the first individual in a family seeking genetic testing, the result of which indicated that he/she was a carrier of a FM (in general, probands with ID) or PM (patients with normal intelligence) (Table 2). A custom-designed program (see Section 2.3) perfectly identified the cases recorded in more than one laboratory, so that they could be taken into account to ascertain the exact number of different ICs. Each new positive PM or FM case not related to others was considered a new family meaning that the number of ICs is the same as the number of families. In total, we have registered 1105 ICs or families and a total of 5062 cases, ICs included, that is, a total of 3957 relatives. It is important to note that we have only included direct relatives at risk by pedigree, excluding all that were not directly related (spouses/partners of individuals with PM or FM, etc.). Some of these families have been included in previous studies [20, 21]. The registry was completed with individuals who underwent molecular genetic testing between 1991 and 2009 who provided informed consent for diagnostic testing.

### 2.2. Molecular Analysis

Molecular analysis of the* FMR1* CGG repeat region was performed in different laboratories following the same method, with an initial screen using PCR analysis of the CGG repeat to exclude males with a normal repeat or females showing two normal alleles, based on the protocol proposed by Fu et al. [22]. In recent years, some of the participating laboratories have used PCR amplification using fluorescent-labelled primers, analysing the size of the amplified fragments on a sequencer ABI310 (Applied Biosystems, Foster City, CA) [14] or PCR amplification followed by detection with nonradioactive methods [23]. Finally, to confirm suspected PMs, mosaic alterations, or FMs, all laboratories used analysis by Southern blot with the StB12.3 probe in DNA double digested with EcoRI + EagI [24].

### 2.3. Data Collection

We created a standardised registry program called ProGGen (an application developed on Lotus Notes) for which Excel files were sent to each collaborating laboratory along with detailed instructions on how to complete each file. The design was developed according to criteria established by clinicians, geneticists, and molecular geneticists, members of the GIRMOGEN network and involved in the genetic diagnosis of FX. Clinical and molecular information comprised the date of birth, year of sampling and study, cellular origin of DNA, sex, number and methylation status of CGG repeats, CGG repeats of the carrier mother/father, mental status, facial dysmorphic features, presence of macroorchidism (in males), and diagnoses of FXPOI or FXTAS. Once received, the completed files were imported into the aforementioned program, which detects and cleans duplicate cases and then carries out the data extraction, processing, and the statistical analysis with its own software.

## 3. Results 

In total, 1200 index cases or families were registered but 95 were shared by two or more laboratories, and hence there were in fact 1105 different families. The total number of individuals entered on the database was 5250, but subtracting 378 shared cases, the total number of different cases—ICs included—was 5062 (Table 1). This yields a total of 3957 females and males tested for each registered IC; that is, the average pedigree size was 3.58. In 268 families we were only able to study. or record, the IC and, at the other extreme, the largest family registered comprises 55 individuals, including the IC.

**Figure 1:**
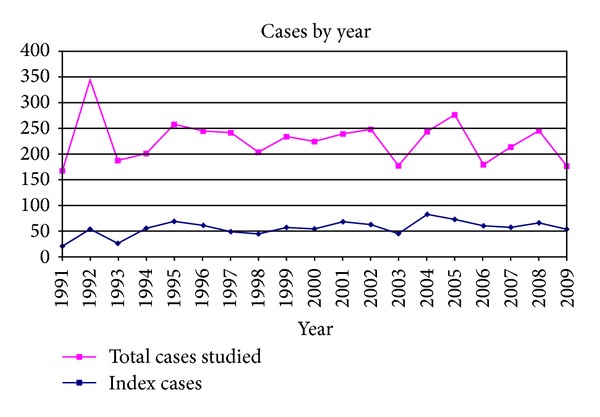
Distribution of cases studied by the year of diagnosis (1991–2009).

Figure 1 shows the distribution of the number of diagnoses per year. The mean number of ICs studied per year was 54.89 and this figure appears to be stable over the years. A peak is observed in the total of individuals studied in 1992 because many of the FX families diagnosed before 1991 by cytogenetic analysis were restudied that year with molecular techniques. There were other smaller peaks in 2004-2005 when families began to be referred for testing because of FXTAS or FXPOI.

Table 2 lists the ICs as a function of the reasons for referral, separating those with ID, developmental problems, and/or autism from those with normal intelligence. As can be seen, 17 ICs were detected among women tested because they had ovarian failure and 13 (10 males and 3 females) were detected among patients with suspected FXTAS. The average age of the IC at diagnosis was 15.8 years (mean calculated with the 840 ICs in which the age at diagnosis was known), being this mean so high due to individuals seeking genetic testing with normal intelligence (Table 2). As for the subgroup of patients with ID, Table 3 classifies them by the age of diagnosis, in 10 year bands, showing that 60.75% of ICs (370/609) were less than 10 years old at diagnosis.

Table 4 shows the results of all the molecular tests performed in the 5062 cases (postnatal and prenatal). Of the total, 969 were FM males and 541 were FM females; 145 were mosaic (96 males and 49 females); 351 were PM males and 1487 PM females, with the remaining 102 cases having IAs. In addition, we have found three deletions, two in prenatal diagnosis (1 female and 1 male). Interestingly, three FM patients also carried a chromosomal abnormality: two males had Klinefelter syndrome and one female (previously reported [25]) had a mosaic Turner syndrome. It is also notable that two FM males were classified as normal from the point of view of intelligence; that is, they were high-functioning men. Finally, one male with a PM also carried a mutation in the FBN1 gene, causing Marfan syndrome.

Among the 271 prenatal diagnoses (Table 5) performed in carrier pregnancies (PM or FM), the mutated allele had been passed to the foetus in 147 cases (147/271 = 54.24%) and the normal allele in 124 (124/271 = 45.75%). The difference is not significant and thus there was no evidence of segregation distortion of the alleles. Similarly, we found an excess of males carrying the chromosome with the FX mutation but the difference was not significant (78/147 = 53.06% versus 68/147 = 46.25%), so there does not appear to be any sex ratio distortion among FX offsprings.

With all these molecular data, we wanted to analyse the instability of the CGG repeat alleles inside the families, and in order not to bias the analysis, we excluded ICs.  Table 6 shows the risk of expansion for females: the results are expressed as the total number of sons and daughters with FMs and PMs in pedigrees, having a mother with PM or IA whose number of CGG repeats is known. These maternal repeat sizes are distributed from ten to ten repeats. In general, there is a growing likelihood of unstable transmissions with increasing repeat size but four points are important to emphasize in interpreting this table: (1) although the great majority of the alleles with more than 110 repeats expanded to a full mutation, the expansion risk was only 100% in two repeat ranges (140–149 and 160–169); (2) in all ranges, except in one, the risk of allelic expansion to FM is higher for male than female offspring (63.6% versus 45.6%; *P* < 0.001); (3) in the IA group, there were 10 cases of expansion to a PM allele; and (4) in the range of the smallest PM alleles (55–59 repeats), there was a 6.4% risk of expansion to a full mutation, with 56 repeats being the smallest allele that expanded to a FM.

Finally, Table 7 shows the instability in the paternal transmissions. It is interesting that there were more expansions than regressions (99 versus 40; *P* < 0.001) but for men with more than 140 repeats all daughters showed regressions of the paternal repeat size.

## 4. Discussion

In clinical practice, the established technique for FX diagnosis is testing for the CGG expansion in the* FM1* gene, what we call molecular genetic diagnosis. In line with this, in Spain all patients with suspected FX are referred for diagnosis to reference centres which use molecular techniques and the results are recorded in local databases that have been operating within these centres since the identification of the* FMR1* gene. In 2006, the 12 largest molecular laboratories (Table 1) decided to bring together the information contained in the individual databases creating a single national registry. The present study summarizes the compilation of molecular data of 5062 individuals from 1105 different FX families from 19 years of diagnostic work. Although FXS is one of the most prevalent genetic disorders, few analyses have been published on large series of individuals belonging to fragile X families, and hence the importance of our report.

From the point of view of clinical practice, we first want to take note of the reasons for referral that resulted in the diagnosis of a new FX family (Table 2). As we can see, 190 ICs (190/1105 = 17.2%) had normal intelligence and they were referred because they had a family history of ID, or because they had an ovarian failure or there was suspicion of FXTAS, and all were found to be carriers of a PM. The diagnosis of new FX families with these selection criteria confirms what has been recommended by the FX American Expert Working Group [26] in the sense that all these reasons for referrals are associated with a high rate of identification of affected individuals and carriers.

Considering the distribution by the age at diagnosis of the 609 ICs with ID for which this age was recorded (Table 3), we observe that only 370/609 (60.75%) were less than 10 years old and as many as 98 ICs were adults (98/609 = 16%). These data can be explained by the fact that in the early years, most studies were performed in adults from institutions for individuals with intellectual disabilities. Table 3 also shows that the IC was a female in 10% of the detected FMs (76 out of 769 cases), a figure similar to others published [17]. Figure 1 shows the distribution of the patients according to the year of diagnosis. Looking at the curve of the ICs, we can see that the number of diagnoses per year has been more or less stable with three small peaks: the first one in 1995, which corresponds to studies in institutions; the second one in 2001, which may correspond to a more widespread awareness of these tests among paediatricians; finally, a third peak in 2004, when tests were introduced for FXPOI and FXTAS. Overall, despite the fact that the number of tests carried out in our centres has increased considerably in recent years (data not shown), it seems that we have reached a ceiling in the detection of new families per year with an incidence of about 50 families. Returning to the cases of ID, we have found no changes in the age at diagnosis of FXS during the last 10 years of records and hence our data indicate that, despite all the information paediatricians and teachers have about FXS, the identification of new cases at younger ages continues to be a challenge [27].

In the event of a positive diagnosis (a new IC), an extension of the molecular study to relatives has always been proposed in Spain, following standard recommendations [4, 17, 26, 28] on cascade testing in the extended family. In total, 3957 females and males were tested (3.58/IC), including 271 prenatal diagnoses (Tables 4 and 5). Table 4 shows that there were more males identified with the full mutation and more females with the premutation as has been reported previously in families [5, 17, 20, 21] and this is attributable to the fact that the great majority of males tested had intellectual disability (Table 2), and their mothers were frequently carriers of the premutation. Furthermore, there is an excess of total females studied (2895 women versus 2152 males) indicating the importance given to the knowledge of the carrier status in females for reproductive purposes. In relation to mosaic cases, although some other research in families [5] indicated that male carriers with a full mutation have mosaic patterns more frequently than females, our results do not corroborate these data (96/1065 = 9% in males versus 49/590 = 8.3% in females).

Regarding the finding of cases of Klinefelter and Turner syndromes, it is interesting to recall that one of the advantages of the FX test is the ability to detect some sex chromosome abnormalities [8], in particular, with the observation either in PCR or Southern blot analysis of two X chromosomes in a male patient. In the large series published by Strom et al. [8] and by Youings et al. [29] not a single case was found of a male patient with both a sex chromosome aneuploidy and a PM or FM allele. They reported a frequency of Klinefelter syndrome of 1 : 702 [8] and 1 : 249 [29] among males studied for FXS. In our registry, 2 of the 969 male patients with FM also had Klinefelter syndrome; a rate intermediate between those found in the aforementioned studies but in accordance with the fact that Klinefelter Syndrome is, by far, the most common sex chromosome aneuploidy. Since the FXS is the most frequent genetic cause of ID, it should not be expected to be rare for the two syndromes to cooccur in a patient.

In relation to prenatal diagnosis, in general, our results corroborate those of previous large series [7, 18, 19] in the sense that there was no evidence of segregation distortion of the allele transmitted nor in the sex segregation. Although it seems there is an excess of male foetuses compared to female foetuses this difference was not statistically significant and is consistent with other reports [8]. We underline that it is important to bear the results in Table 5 in mind for genetic counselling, because the overall cases recorded (Table 4) correspond to a retrospective analysis of fragile X families that may suffer from ascertainment bias, while prenatal data are always considered prospective and not subject to this source of error [19].

Concerning the mutation expansion risks for females with IA or PM alleles (Table 6), our work also confirms previous studies [7, 8, 18, 19] in the sense that the instability of PM alleles increases with the size of alleles. It has been suggested that the lower expansion rate of the smaller PMs is due to the presence of an AGG sequence in the middle of the CGG repeats that creates an anchor protecting against expansion [7]. Since our molecular data were recorded with the size alone, we have no way of knowing which cases have AGG sequences. In any case, there are some differences with the previous studies that we want to highlight. First, in mothers with 50 to 54 repeats (IAs), they expanded to a PM allele in 10 cases and in the range of the smallest PM alleles, 6.4% of alleles of 55 to 59 repeats expanded in a single meiosis to a FM allele [30, 31]. Furthermore, for ICs (not included in Table 6), the smallest maternal allele observed to expand to a FM allele contained 56 repeats. Hence, in our series the risk for alleles of <59 repeats is somewhat higher than in other published series [8, 18, 19] and recommendations for prenatal testing must be established in that range. Furthermore, in our study, the alleles of up to 79 repeats expanded more frequently to PM than to FM alleles and the highest rate of full mutation expansion appeared in mothers with 90 repeats or more, whereas Nolin et al. [19] observed this higher rate for mothers with 80 repeats or more. We also observed that, in all ranges except one (110–119 repeats), the risk of expansion to a FM is higher for male than female offspring (63.6% versus 45.6%; *P* < 0.001), and that provides strong evidence that the transition from PM to FM is a postzygotic event; that is, it occurs after fertilization of the carrier oocyte [5]. These patterns are of vital importance for genetic counselling.

Evidence from other triplet-repeat disorders also points to postzygotic events that contribute to these differences in the repeat instability. They have also been observed in maternal transmissions of CAG repeats in Huntington disease, with a tendency for expansion in male offspring and contractions in female offspring [32]. In myotonic dystrophy although contractions of the CTG repeats are much less frequent than expansions, they are more frequently transmitted by males [33]. In our study, contractions also occur in maternal transmissions but only in a 2.6% of them, with no differences between male and female offsprings. By the contrary, it is interesting that, for carrier men, there were more transmissions with expansions than contractions (99 versus 40; *P* < 0.001) but, for men with more than 140 repeats, all daughters showed regressions in size—including four daughters with a PM whose fathers had >200 repeats—indicating once more that expansions are a postzygotic event. Thus, events occurring after fertilisation may play a role in determining repeat size in FX as it does in other expansion disorders [32], and these events may be heritable as we suggested in a previous work the possible existence of an intrafamilial effect [12].

## 5. Conclusions

In conclusion, our data fully validate the use of molecular genetic tests for fragile X in clinical practice. It also supports and completes previous studies, adding more evidence and additional data that may be useful for the purposes of genetic and reproductive counselling.

## Figures and Tables

**Table 1:** Participating laboratories/centres and their total number of individuals registered.

Laboratories/centres	Number of cases in each centre	Shared cases	Number of IC or families in each centre
Hospital Clínic (Barcelona)	1.623	116	405
Hospital V. Arrixaca (Murcia)	1.001	9	104
Hospital Universitario Cruces (Basque Country and Navarra)	744	24	125
Hospital La Fe (Valencia)	608	33	108
Hospital Ramón y Cajal (Madrid)	403	1	133
C. Sanitaria Parc Taulí (Sabadell-Catalonia)	225	117	97
Hospital Virgen Macarena (Sevilla)	184	29	78
Hospital Son Espases (Illes Balears)	149	12	35
Hospitales Miguel Servet and Lozano Blesa (Zaragoza)	101	3	32
Hospital regional de Málaga (Málaga and Sevilla)	82	27	21
IBGM, Universidad de Valladolid (Castilla-León)	76	4	35
F. Jiménez Díaz (Madrid)	54	1	27

Total number of individuals studied	5062^a^	378	1105^b^

^a^The total sum is 5250 cases, but subtracting the 188 shared cases yields a total of 5062 different cases.

^b^The total sum of families is 1200 families, of which 95 were shared, giving a total of 1105 different families.

**Table 2:** Medical indication for referral.

	Number of males	Number of females	Total number
Patients with ID			
With family history of ID			
GDD, ID, and autism (all ages)	97	8	105
Studies in ID institutions	22	0	22
With no family history of ID			
Children with GDD and or autism	461	55	516
ID in young patients and adults	113	13	126
Patients with normal intelligence			
With family history of ID			
Relative of a patient with FXS diagnosed in another centre	30	112	142
Individual with history of ID with unknown aetiology in his/her family	2	16	18
With no family history of ID			
POI and/or menopause	0	17	17
FXTAS	10	3	13
Unknown reason to be studied	106	40	146

Total	841	264	1105

**Table 3:** Distribution of the 769 ICs with ID, by the age of the diagnosis of FXS.

Age of diagnosis	0–9	10–19	20–29	30–39	40–49	50–59	60+	Age unknown	Total
Number of males	333	125	43	26	7	5	4	150	693
Number of females	37	16	5	5	2	0	1	10	76

Total	370	141	48	31	9	5	5	160	769

**Table 4:** Results of the 5,062 molecular diagnostic tests performed in the 1105 different families (ICs and prenatal cases included).

	FM	Mosaics	PM	IA	Deletions	Normal	Total
Number of males	969	96	351	42	2	692	2152
Number of females	541	49	1487	60	1	757	2895

Total	1510	145	1839^a^	102	3	1458^a^	5057^b^

^a^The sex was unknown in 1 premutation and in 9 normal cases. All were prenatal cases (see Table 5).

^b^In 5 cases, the molecular status was not recorded in the database.

**Table 5:** Molecular results of the prenatal studies performed in pregnant women carriers of an FM or PM.

	FM	Mosaics	PM	Deletions	Total fragile-X	Normal-X	Total
Number of males	61	1	15	1	78	64	142
Number of females	51	2	14	1	68	51	119

Total	112	3	30^a^	2	147^a^	124^a^	271^a^

^a^The sex was unknown in 1 premutation and in 9 normal cases.

**Table 6:** Mutation expansion risks for carrier females of a PM or IA (including PN cases but not ICs).

Maternal repeat size	Male offspring			Female offspring			Total		
	Number of premutation	Number of full mutation	% full mutation	Number of PM	Number of. full mutation	% full mutation	Number of PM	Number of full mutation	% full mutation
44–49	0	0	0.0	0	0	0.0	0	0	0.0
50–54	4	0	0.0	6	0	0.0	10	0	0.0
55–59	11	0	0.0	33	3	8.3	44	3	6.4
60–69	22	7	24.1	60	11	15.5	82	18	18.0
70–79	26	24	48.0	53	30	36.1	79	54	40.6
80–89	18	31	63.3	36	30	45.5	54	61	53.0
90–99	2	21	91.3	12	28	70.0	14	49	77.8
100–109	2	22	91.7	5	22	81.5	7	44	86.3
110–119	1	7	87.5	0	15	100.0	1	22	95.7
120–129	0	10	100.0	2	7	77.8	2	17	89.5
130–139	0	13	100.0	1	12	92.3	1	25	96.2
140–149	0	3	100.0	0	5	100.0	0	8	100.0
150-159	0	3	100.0	1	2	66.7	1	5	83.3
160–169	0	5	100.0	0	7	100.0	0	12	100.0
170–199	1	6	85.7	0	3	100.0	1	9	90.0

Total	87	152^a^	63.6^a^	209	175^a^	45.6^a^	296	327	52.5

^a^Differences in the risk of expansion from PM to FM between males and females offsprings has statistical significance (*P* < 0.001).

**Table 7:** Paternal transmissions to their daughters.

Paternal repeat size	Number with the same number of repeats	Number with regression	Median of the repeats average difference	% regression	Number with expansion	Median of the repeats average expansion	% expansion
50–59	11	1	5.0	3.4	17	11.6	58.6
60–69	8	6	2.3	14.3	28	13.1	66.7
70–79	3	4	7.8	15.4	19	17.4	73.1
80–89	12	3	7.0	9.1	18	32.1	54.5
90–99	5	6	22.0	33.3	7	32.4	38.9
100–109	0	3	19.3	37.5	5	35.8	62.5
110–119	1	3	15.3	37.5	4	26.8	50.0
120–129	1	3	39.0	60.0	1	17.0	20.0
130–139	1	0	0	0.0	0	0	0.0
140–149	0	3	26.7	100.0	0	0	0.0
150-159	0	0	0	0.0	0	0	0.0
160–169	0	0	0	0.0	0	0	0.0
170–179	0	0	0	0.0	0	0	0.0
180–198	0	4	73.5	100.0	0	0	0.0
190–199	0	0	0	0.0	0	0	0.0
>200	0	4^a^	471.0	100.0	0	0	00.0

Total	42	40		22.1	99		54.7

^a^These 4 daughters had PMs, with their fathers being 2 high functioning males.
